# Transcriptomics of Canine Inflammatory Mammary Cancer Treated with Empty Cowpea Mosaic Virus Implicates Neutrophils in Anti-Tumor Immunity

**DOI:** 10.3390/ijms241814034

**Published:** 2023-09-13

**Authors:** Lucia Barreno, Natalia Sevane, Guillermo Valdivia, Daniel Alonso-Miguel, María Suarez-Redondo, Angela Alonso-Diez, Steven Fiering, Veronique Beiss, Nicole F. Steinmetz, Maria Dolores Perez-Alenza, Laura Peña

**Affiliations:** 1 Department of Animal Medicine, Surgery and Pathology, Mammary Oncology Unit, Veterinary Teaching Hospital, Veterinary Medicine School, Complutense University of Madrid, 28040 Madrid, Spain; lbarreno@ucm.es (L.B.); edgargva@ucm.es (G.V.); danialon@ucm.es (D.A.-M.); marsuare@ucm.es (M.S.-R.); angalo02@ucm.es (A.A.-D.); mdpa@ucm.es (M.D.P.-A.); laurape@ucm.es (L.P.); 2 Department of Animal Production, Complutense University of Madrid, 28040 Madrid, Spain; nsevane@ucm.es; 3 Department of Microbiology and Immunology, Geisel School of Medicine at Dartmouth, Lebanon, NH 03756, USA; 4 Dartmouth Cancer Center, Geisel School of Medicine at Dartmouth, Lebanon, NH 03756, USA; 5 Department of NanoEngineering, University of California San Diego, La Jolla, CA 92093, USA; verobeiss@googlemail.com (V.B.); nsteinmetz@ucsd.edu (N.F.S.); 6 Department of Radiology, University of California San Diego, 9500 Gilman Dr., La Jolla, CA 92093, USA; 7 Department of Bioengineering, University of California San Diego, 9500 Gilman Dr., La Jolla, CA 92093, USA; 8 Moores Cancer Center, University of California San Diego, 9500 Gilman Dr., La Jolla, CA 92093, USA; 9 Center for Nano-ImmunoEngineering, University of California San Diego, 9500 Gilman Dr., La Jolla, CA 92093, USA; 10 Institute for Materials Discovery and Design, University of California San Diego, 9500 Gilman Dr., La Jolla, CA 92093, USA; 11 Center for Engineering in Cancer, Institute for Engineering in Medicine, University of California San Diego, 9500 Gilman Dr., La Jolla, CA 92093, USA

**Keywords:** canine mammary cancer model, intratumor immunotherapy, gene expression profile, cowpea mosaic virus, neutrophils, tumor microenvironment

## Abstract

Canine inflammatory mammary cancer (IMC) is a highly aggressive and lethal cancer in dogs serving as a valuable animal model for its human counterpart, inflammatory breast cancer (IBC), both lacking effective therapies. Intratumoral immunotherapy (IT-IT) with empty cowpea mosaic virus (eCPMV) nanoparticles has shown promising results, demonstrating a reduction in tumor size, longer survival rates, and improved quality of life. This study compares the transcriptomic profiles of tumor samples from female dogs with IMC receiving eCPMV IT-IT and medical therapy (MT) versus MT alone. Transcriptomic analyses, gene expression profiles, signaling pathways, and cell type profiling of immune cell populations in samples from four eCPMV-treated dogs with IMC and four dogs with IMC treated with MT were evaluated using NanoString Technologies using a canine immune-oncology panel. Comparative analyses revealed 34 differentially expressed genes between treated and untreated samples. Five genes (*CXCL8*, *S100A9*, *CCL20*, *IL6*, and *PTGS2*) involved in neutrophil recruitment and activation were upregulated in the treated samples, linked to the IL17-signaling pathway. Cell type profiling showed a significant increase in neutrophil populations in the tumor microenvironment after eCPMV treatment. These findings highlight the role of neutrophils in the anti-tumor response mediated by eCPMV IT-IT and suggest eCPMV as a novel therapeutic approach for IBC/IMC.

## 1. Introduction

Human inflammatory breast cancer (IBC) and canine inflammatory mammary cancer (IMC) are highly metastatic and deadly types of mammary cancer, commonly triple-negative, that lack effective therapies in both species [1,2,3]. IBC/IMC are an uncommon type of breast/mammary cancer with unusual specific genetic, pathogenic, and clinical characteristics [3,4,5]. The criterium for histological diagnosis of IBC/IMC is the neoplastic embolization of superficial dermal lymphatic vessels, which blocks lymphatic drainage and causes distinctive clinical features: mainly distinct diffuse inflammation and edema of the skin [3,4]. Intensive research efforts are ongoing to discover effective therapies for invasive and metastatic IBC [6,7]; however, optimal results have not been achieved [7]. The challenge in finding effective therapies for IBC is, in part, due to the lack of suitable experimental models to understand the aggressive behavior and mechanisms of IBC/IMC, and the limited knowledge of in vivo IBC/IMC biology. Most of our current understanding comes from studying cell lines and patient-derived models in mice lacking a functional immune system [8].

Dogs are genetically outbred animals with cancer prevalence, tumor type, and tissue origin that are similar to human cancer. In contrast to rodent cancer models, canine patients are outbred, long-lived, large patients that spontaneously develop cancers in a syngeneic environment in the presence of an intact immune system, thereby replicating the biology and heterogeneity of human disease [9,10,11]. Numerous epidemiologic, clinical, histopathological, and ultrastructural characteristics are shared by IBC and IMC, making IMC the only identified animal model that recapitulates the overall biology of IBC [12,13,14]. Canine IMC is a unique and excellent preclinical therapeutic model to evaluate the efficacy of new therapies for IBC [15].

Immunotherapy is a promising therapeutic strategy for cancer, given recent clinical advances in treating other human cancers, such as melanoma and lung cancer, that have demonstrated impressive clinical responses [16,17,18]. The activation of the immune system to recognize tumor antigens for cancer elimination [19] is an encouraging and novel therapeutic approach, although minimal progress has been made in the immunotherapy for breast cancer (BC) [20,21]. Immunotherapy with nanoparticles is an actively explored and promising area in oncology [22].

Our previous studies demonstrated the capacity of plant-based virus-like nanoparticles (VLPs) from empty cowpea mosaic virus (eCPMV) to stimulate anti-tumor immune responses and to enhance outcomes in different syngeneic murine BC tumor models [23,24] and in dogs with spontaneous IMC [25], with no side effects or toxicities. The immunotherapy involves the in situ administration of VLPs, which exhibit good tumor retention given the nanoparticle characteristics (measuring 30 nm in size) and are stable and non-toxic. CPMV can infect some plants but does not infect animal cells, while eCPMV lacks viral RNA, so is completely non-infectious [23]. Previous studies have demonstrated that the capsid of eCPMV is recognized by Toll-like receptors (TLR2 and TLR4) at the plasma membrane, triggering downstream signaling cascades involving the adaptor protein MyD88, ultimately activating the NF-κB pathway [26] in order to induce pro-inflammatory cytokines and anti-tumor efficacy. In this manner, the intratumor immunotherapy (IT-IT) strategy involves the direct manipulation of identified tumors to overcome the local tumor-mediated immunosuppression and, subsequently, stimulate systemic anti-tumor immunity [27].

The present study Is a continuation of a previous one conducted by our research group [25], in which eCPMV IT-IT stimulated a potent local leukocyte activation, induced a strong neutrophilic tumor infiltration, reduced tumor volume, and improved survival and quality of life in dogs with IMC, without adverse effects.

Transcriptomic analyses are powerful tools for detecting genes that influence the immune response. In BC, adaptive immune programs play diverse roles depending on the cellular infiltration found in each tumor [28], which is further complicated by heterogeneous genomic expression and genome instability [29,30]. There are no previous studies on the transcriptomic profile of dogs with IMC before or after treatment with eCPMV nanoparticles. Therefore, comparing the transcriptomic profiles in treated and untreated IMC samples can shed light on the complex gene networks involved and the improved clinical outcome of treated dogs with IMC.

In this study, an innovative NanoString nCounter Canine Immuno-oncology (IO) panel was used to evaluate RNA expression, active signaling pathways, and immune cell subtyping in tumors of female dogs diagnosed with IMC and treated with eCPMV IT-IT versus untreated IMC female dogs (only receiving medical therapy, MT) in order to characterize the immune response induced by the treatment. This advanced technology enables the analysis of numerous immune-related genes using RNA extracted from formalin-fixed paraffin-embedded (FFPE) tissues. These experiments also investigated the association between differential gene expression after eCPMV IT-IT and the clinical/cellular characterization obtained via flow cytometry, cytokine, histology, and immunohistochemistry assays in our previous study [25]. Our data show that eCPMV IT-IT induces strong transcriptional changes in the neoplastic tissue, which modify host immunity to promote potent neutrophil recruitment and activation against the tumor.

## 2. Results

### 2.1. Differentially Expressed Genes in Tumor Samples after eCPMV Treatment

**Figure 1:**
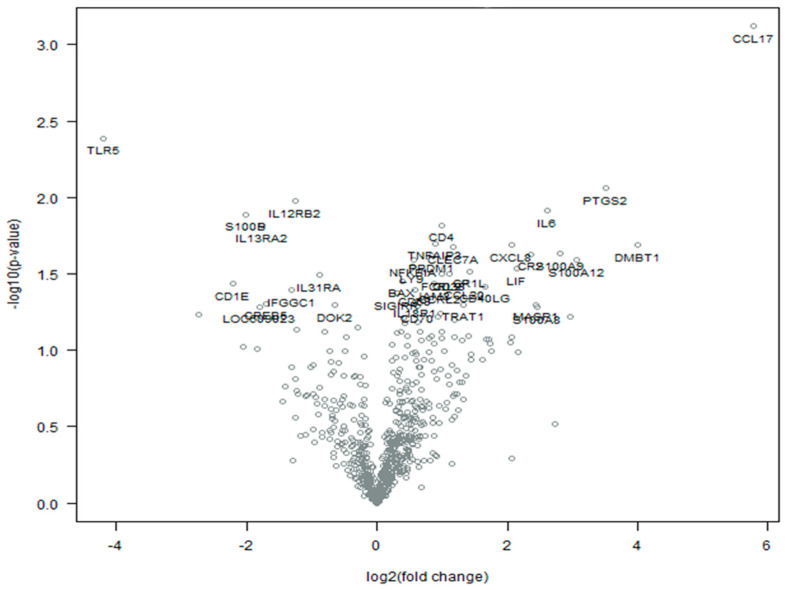
Volcano plot of differentially expressed genes between treated and untreated IMC samples with eCPMV immunotherapy. Thirty-four genes were significantly differentially expressed in IMC samples after eCPMV in situ immunotherapy versus untreated IMC samples (*p* value < 0.05), 26 upregulated with a positive log2FC and 8 downregulated with a negative log2FC.

A total of 696 of the 780 immuno-oncology genes included in the NanoString nCounter technology passed QC and were included in the downstream analysis (Table S3). The comparison of gene expression profiles between the eCPMV-treated and -untreated samples revealed 34 significantly differentially expressed genes (DEGs) (*p* < 0.05), 26 of them upregulated (positive log2FC) and 8 downregulated (negative log2FC) in treated samples (Table 1; Figure 1).

Most of the DEGs observed in the IMC-treated samples were involved in chemokine and cytokine signaling pathways, including *CCL17*, *PTGS2*, *IL12RB2*, *IL6*, *S100B*, *CD4*, *IL13RA2*, *CXCL8*, *S100A12*, *NFKBIA*, *LIF*, *IL31RA*, *CCL20*, *CD40LG*, *SIGIRR*, and *IL18R1*. Among them, *CCL17*, *S100B*, and *SIGIRR* appeared to be specifically associated with the chemokine and cytokine signaling pathways. Furthermore, the expression of *CCL17* mRNA showed the highest increase after treatment (log2FC = 5.77). On the other hand, the lowest downregulation value was displayed by *TLR5* (log2FC = −4.21), a member of the Toll-like receptor family (TLR).

### 2.2. Pathway Analyses

The expression data were analyzed using the KEEG pathway analysis tool from DAVID. Of the 26 upregulated genes in the treated group, 25 were included in the analysis, resulting in a total of 37 pathways (Table S4). Among these pathways, the most relevant ones, which included five or more genes, were the interleukin 17 (IL-17) signaling pathway (*NFKBIA*, *IL6*, *CXCL8*, *CCL20*, *TNFAIP3*, *PTGS2*, *CCL17*, *S100A9*), and cytokine–cytokine receptor interaction (*IL6*, *CD4*, *CD40LG*, *CXCL8*, *CCL20*, *LIF*, *CCL17*, *IL18R1*), followed by the tumor necrosis factor (TNF) signaling pathway (*NFKBIA*, *IL6*, *CCL20*, *LIF*, *TNFAIP3*, *PTGS2*, *IL18R1*), viral protein interaction with cytokine and cytokine receptors (*IL6*, *CXCL8*, *CCL20*, *CCL17*, *IL18R1*), and the nuclear factor-kappaB (NF-κB) signaling pathway (*NFKBIA*, *CD40LG*, *CXCL8*, *TNFAIP3*, *PTGS2*). It is worth pointing out three other upregulated pathways, given their previous implication in key processes elicited after eCPMV IT-IT: the chemokine signaling pathway (*NFKBIA*, *CXCL8*, *CCL20*, *CCL17*), the Toll-like receptor signaling pathway (*NFKBIA*, *IL6*, *CXCL8*), and Th17 cell differentiation (*NFKBIA*, *IL6*, *CD4*). On the other hand, from the eight downregulated genes in the treated group, seven were included in the analysis and three pathways were retrieved (Table S4), with the decreased JAK-STAT signaling pathway (*IL13RA2*, *IL12RB2*) being the most interesting.

### 2.3. Cell Type Profiling

**Figure 2:**
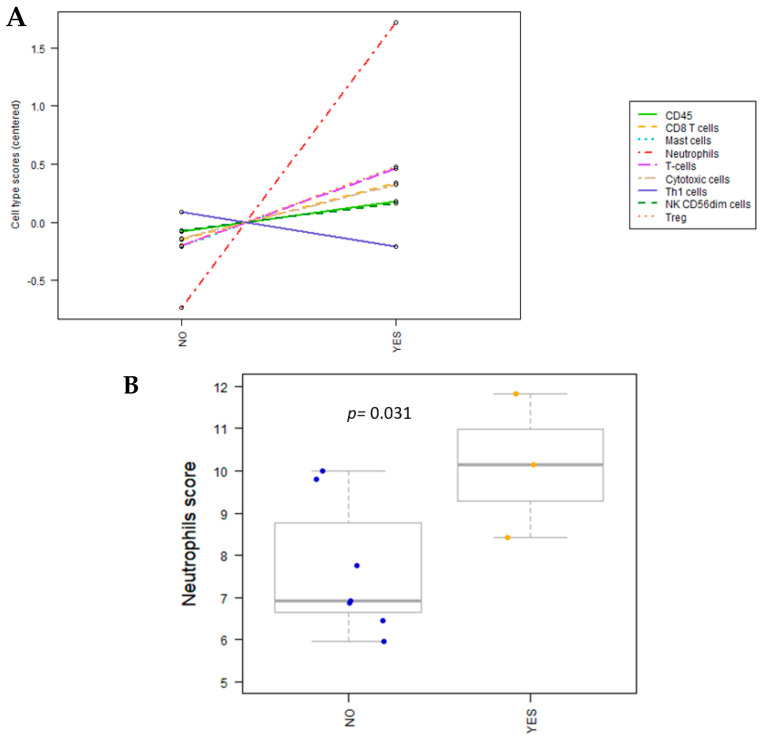
(**A**) Cell type profiling between IMC samples treated and untreated with eCPMV immunotherapy (NO: untreated IMC samples; YES: eCPMV-treated IMC samples). IMC-treated samples showed a remarkably higher neutrophil population than untreated samples among all the rest of the cell population. (**B**) Neutrophil subpopulations were significantly increased (*p =* 0.031) in treated IMC samples versus untreated IMC samples (*p* values estimated using Student’s *t*-test).

The nCounter software nSolver 4.0 used for canine NanoString analyses includes a specific cell type profiling analysis that can detect the abundance of immune cell populations by evaluating specific transcripts. This approach employs genes that have been previously identified as characteristic of various cell populations to measure the abundance of immune cellular populations in the TME. The IMC-treated samples showed a significantly higher proportion of neutrophils (*p =* 0.031) than the untreated samples (Figure 2A,B and Table 2). No other significant changes in immune cell populations were found with a *p* value < 0.05. However, there was a potential increase in T regulatory (Treg) cells and overall T cells, with *p* values < 0.1.

## 3. Discussion

In this study, we conducted a comprehensive evaluation of the transcriptomic profile and immune response in female dogs with IMC who received intratumoral eCPMV immunotherapy, compared to control untreated patients. By utilizing the NanoString nCounter Canine IO panel, we were able to analyze RNA expression and classify immune cell subtypes, providing valuable insights into the immune response following treatment. This approach allowed us to gain a deeper understanding of the signaling pathways involved in the anti-tumor immune response triggered by eCPMV nanoparticles, as well as identify potential candidate genes contributing to leukocyte activation. Our findings demonstrated significant transcriptional changes in the TME following eCPMV nanoparticle intratumoral immunotherapy. This is the first study in dogs with IMC to assess the impact of in situ eCPMV immunotherapy on the immune response using transcriptomics.

We identified 34 significant DEGs between treated and untreated IMC samples: 26 genes were upregulated and 8 genes downregulated in the treated group. The upregulated DEGs were primarily involved in signaling pathways related to chemokines and cytokines, indicating the activation of immune responses against the tumor. Among these, the IL-17 signaling pathway and cytokine–cytokine receptor interactions were particularly relevant, as they play crucial roles in immune responses and inflammation, suggesting their contribution to the anti-tumor effects triggered by eCPMV immunotherapy treatment.

### 3.1. The IL-17 Signaling Pathway’s Role in the Immune Response Triggered by eCPMV Immunotherapy

**Figure 3:**
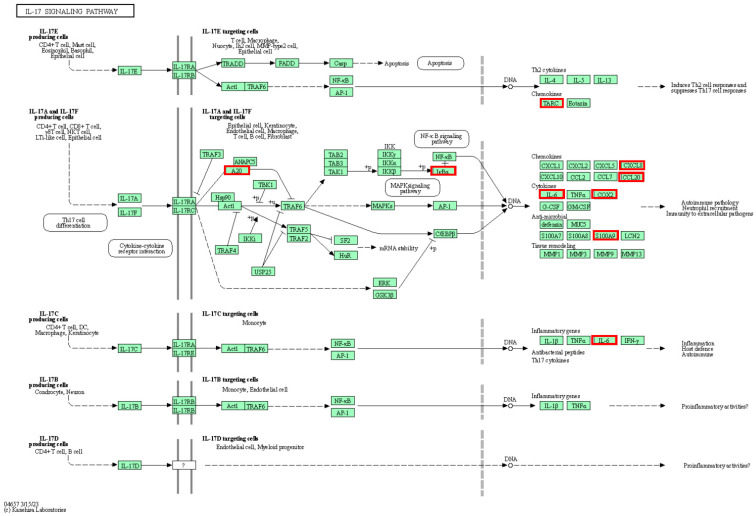
IL-17 signaling pathway (cfa04657). Genes upregulated after treatment with eCPMV nanoparticles in IMC tumors are highlighted in red. KEGG IDs—official gene symbol equivalence: A20—TNFAIP3; *IκBα*—NFKBIA; COX2—PTGS2; TARC—CCL17.

The IL-17 signaling pathway, which encompasses eight upregulated genes (*NFKBIA*, *IL6*, *CXCL8*, *CCL20*, *TNFAIP3*, *PTGS2*, *CCL17*, *S100A9*), includes five loci (*CXCL8*, *S100A9*, *CCL20*, *PTGS2*, and *IL6*) involved in the recruitment of immune cells, particularly neutrophils, to sites of inflammation (Figure 3). The *CXCL8* gene encodes interleukin-8 (IL-8), which is a major cytokine that attracts and activates human neutrophils [31].

Intratumor *CXCL8* expression is believed to play a critical role in regulating the recruitment of neutrophils into the TME [32]. In our previous study in dogs with IMC, we observed that eCPMV immunotherapy led to elevated blood levels of mature and immature neutrophils, transient increases in IL-8 levels, and a significant infiltration of neutrophils into the TME [25]. These findings suggest that eCPMV immunotherapy also has the potential to modulate neutrophil response in the TME at a transcriptomic level.

*S100A9* gene, a member of the S100 calcium binding protein family, also known as calprotectin, is abundantly expressed in the cytosol of neutrophils and monocytes. Itcan induce degranulation of neutrophils, through the increased surface exposure of proteins found in secretory vesicles and gelatinase granules [33,34]. The increased expression of *S100A9* in IMC-treated patients with eCPMV may contribute to neutrophil release of the enzyme myeloperoxidase (MPO) and corroborates the higher expression of this enzyme observed by immunohistochemistry (IHC) in our previous results [25]. In a recent study, it was shown that the *S100A8*/*S100A9* complex acts as a damage-associated molecular pattern (DAMPs) molecule, activating TLR4 and initiating a signaling pathway that promotes the movement of the protein MyD88 from the cytoplasm to the cell membrane receptor complex, enhancing NF-κB-dependent transcriptional activity [35]. The present study identifies a substantial number of crucial genes implicated in the NF-κB signaling pathway, that play essential roles in the immune response, supporting the relevance of future development of anti-cancer therapies involving this pathway in combination with eCPMV. Furthermore, previous studies have demonstrated that the capsid of eCPMV is recognized by TLR2 and TLR4 at the plasma membrane, triggering downstream signaling cascades involving the adaptor protein MyD88, ultimately activating the NF-κB pathway [26]. This data supports the interpretation that *S100A9* in IMC-treated animals may contribute to neutrophil degranulation, *TLR4* activation, and enhanced NF-κB-dependent transcriptional activity through MyD88, and promotion of an anti-tumor immune response. Additionally, the upregulation of S100A12, another member of the S100 calcium binding protein family, after eCPMV treatment in this study suggests its regulatory effect on cytoskeletal components involved in various neutrophil activities and their migration [36].

**Figure 4:**
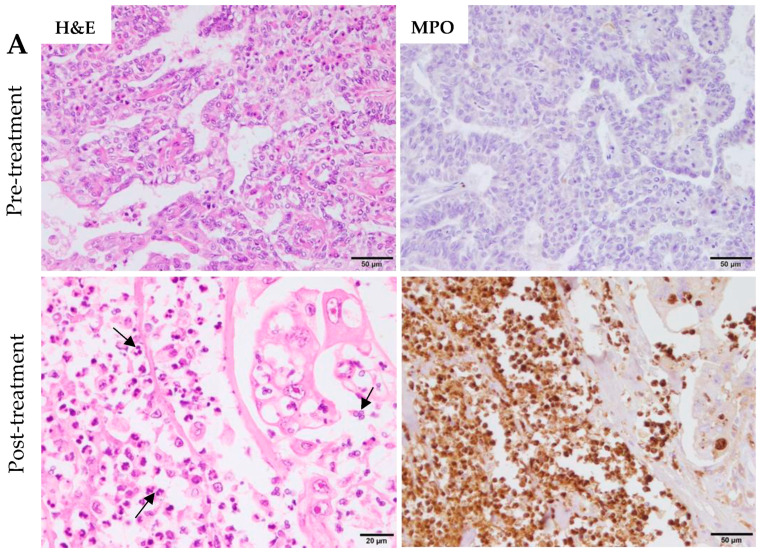

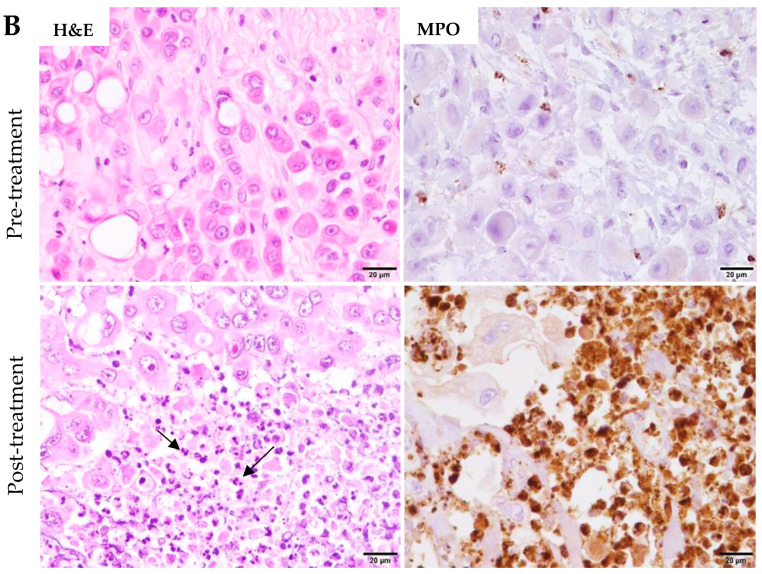
In situ eCPMV immunotherapy in P1 (**A**) and P6 (**B**) induced a high neutrophilic infiltration and associated tumor cell death (necrosis) in post-treatment as compared with pre-treatment tumor samples as indicated by H&E and myeloperoxidase (MPO) staining. Black arrows show multilobulated neutrophil subpopulation.

These findings at transcriptional level agree with our previous studies which established a strong neutrophilic tumor infiltration associated with necrosis, upholding neutrophils as drivers of tumor cell death [25]. In Figure 4A,B demostrated the large neutrophilic infiltration and associated tumor cell death (necrosis) in post-treatment samples as compared with pre-treatment tumor samples in two patients by hematoxylin and eosin (H&E) and MPO staining. In our previous results published [25], MPO expression was significantly higher in post- treatment than in pre-treatment tumor samples.

In this study, the cell type profiling analysis showed a significant increased proportion of neutrophils in the treated versus untreated samples, corroborating their potential involvement in the anti-tumor immune response after eCPMV treatment. Although not reaching statistical significance, the observed changes in Treg lymphocytes and T cells highlight the overall modulation of immune cell populations following eCPMV IT-IT.

Recent studies on the role of tumor-associated neutrophils (TANs) in cancer biology indicate two TAN subpopulations (N1 and N2) having a dual role in tumor inhibition (N1) and progression (N2) [37,38]. The ability to distinguish between these neutrophil subpopulations is critical to understanding the pro- versus anti-tumor effects of TANs. TANs are influenced by signals in the TME such as the specific profile of chemokines, changing their phenotypic or functional plasticity between subpopulations [37,38,39,40]. The transcriptional profiles of the N1 (anti-tumor) and N2 (pro-tumor) TANs are currently under investigation [37,41]. The N1 subpopulation upregulates genes such as *CCL2*, *CCL3*, *CXCL10*, and *CCL7* and downregulates *CCL17*, *CXCL1*, and *CXCL14* [37]. Conversely, the N2 subpopulation upregulates a range of cytokines such as *CCL17* and *CCL5* [37,41].

Although the methodology of the present study does not allow for the separate detection of TAN subpopulations, it is worth noting that the upregulation of the S100A9 gene, as observed in our study, has been shown to enhance the chemotactic and enzymatic activity of the N1 (anti-tumor) subpopulation [42], which suggests that the upregulation of *S100A9* could play an important role in the functional activity of N1 cells after eCPMV treatment. We have previously demonstrated that eCPMV treatment in dogs with IMC [25] dramatically increases TANs as the major driver of the anti-tumor immune response. Further studies are necessary to determine if the N1 subpopulation predominates among neutrophils in the TME after eCPMV treatment.

On the other hand, the high transcriptional overexpression of *CCL17*, also called *TARC*, seems to have a complex effect on tumor immunity. Some studies have associated CCL17 with the N2 tumor-promoting subtype due to its role in immune suppression mediated by regulatory T cells [37,43]. However, CCL17 is also expressed specifically by neutrophils and macrophages and plays a significant role in the alternative cross-priming of dendritic cells (DCs), enabling them to trigger CD8^+^ T lymphocyte responses against tumor antigens [44]. The response of CD8^+^ T cells involves the interaction between activated NK cells and DCs, leading to the production of CCL17, which attracts naïve CD8^+^ T lymphocytes. Increased expression of CD8^+^ granzyme B^+^ T cells has been correlated with higher transcript levels of *CCL17*, and higher serum levels of CCL17 were also associated with progression-free survival in advanced melanoma patients undergoing dendritic-cell-based immunotherapy [45]. Additionally, high serum levels of CCL17 have been associated with improved survival in human melanoma patients [46].

In a murine breast cancer model, the introduction of an adenoviral vector encoding *CCL17* led to significant tumor regression and the generation of specific immunity in the TME [47], supporting a crucial role of this chemokine in the anti-tumor immune response.

At this point, it is interesting to remark that IBC/IMC is a rare and very special type of cancer with unique genetic, pathogenic, and clinical features [3,4,5]. In our study, the longest survival response to eCPMV therapy resulted in a patient (P1) that, interestingly, exhibited a remarkably high number of *CCL17* RNA transcripts after treatment compared to the other eCPMV-treated patients. These results correlate with the significantly higher changes in the peripheral blood of CD8^+^ granzyme B^+^ T cells caused by eCPMV immunotherapy in our previous study of eCPMV treatment for dogs with IMC [25]. Further investigation is warranted to understand the clinical significance of this chemokine in the context of eCPMV immunotherapy.

After eCPMV treatment, the expressions of *IL6* and *CD4* genes were found to be significantly increased. These genes are involved in the differentiation of Th17 cells (Table S4), which produce IL17A and IL17F. Th17 cells play a crucial role in tumor immunity by attracting DCs and activating CD8^+^ T cells to fight against the tumor [48]. In our previous study, an increase in peripheral blood of CD8^+^ granzyme B^+^ T cells was observed in eCPMV-treated patients, which aligns with the role of Th17 cells in generating cytotoxic CD8^+^ T cells for tumor defense [25]. These findings highlight the potential importance of the IL17 signaling pathway in the immune response and its ability to modulate the anti-tumor immune response following treatment.

Interestingly, it has been recently shown that mature neutrophils could have an additional function as professional antigen-presenting cells (APCs) capable of inducing Th17 differentiation [49]. In our current study, genes associated with Th17 differentiation and IL17 signaling pathways, which may potentially recruit neutrophils (Table S4; Figure 3), were significantly upregulated following eCPMV IT-IT. This suggests that the increased proportion of neutrophils observed in IMC-treated patients (Figure 2) could shift their non-specific role to a specific immune action by behaving as APCs after nanoparticle inoculation. Consequently, this process could establish a cycle in which neutrophils participate in antigen presentation and induce Th17 cell differentiation, modulating the anti-tumor immune response after treatment.

The upregulation of *CCL20* and *PTGS2* after eCPMV treatment in our study has interesting implications. *CCL20* can contribute to tumor progression by modulating the functions and phenotype of immune cells in the TME, particularly by inducing PD-L1 expression on neutrophils [50,51]. *CCL20* is a potent chemoattractant of neutrophils to the TME [50,52]. In our study, increased expression of *CCL20* in IMC-treated samples may be associated with the crucial involvement of the IL-17 signaling pathway in the immune response after eCPMV immunotherapy (Figure 3). It has been demonstrated that the IL-17 signaling pathway can increase *CCL20* transcription [53]. Another upregulated gene, *PTGS2* (Log2FC = 3.51), also known as cyclooxygenase-2 (COX-2), is the key enzyme in prostaglandin biosynthesis. This gene is highly expressed in the context of inflammatory responses and plays an important role in BC by influencing immune responses among other functions [54]. In this manner, the anti-tumor inflammatory response triggered by eCPMV treatment could potentially be the underlying cause of its overexpression. *PTGS2* (COX-2) might shift its primary pro-tumor role to promote a potent immune response, thereby acquiring an anti-tumor effect. The pleiotropic nature of *PTGS2* encompasses a wide range of effects, including the stimulation of angiogenesis and tumor cell proliferation [54]. Despite its role in tumor progression, a survival analysis revealed that BC patients with a high expression level of *PTGS2* had a longer survival time after established treatments [55]. However, given that *PTGS2* is generally known as a potent promoter of carcinogenesis, it is necessary to conduct further studies to determine its role in IMC patients following eCPMV IT-IT.

### 3.2. Other Upregulated Immune Pathways and Genes Triggered by eCPMV Immunotherapy

This study identifies crucial genes involved in the NF-κB signaling pathway, viral protein interactions with cytokine and cytokine receptors, the TNF signaling pathway, and the Toll-like receptor signaling pathway, all of which play essential roles in immune responses. Previous studies conducted on mice have demonstrated that the recognition of eCPMV viral capsids induces a downstream signaling cascade through TLRs 2 and 4, supporting the significance of these pathways [35].

In our analysis, we observed an upregulation of *DMBT1* (Log2FC = 4), a tumor suppressor gene known to inhibit cell proliferation and survival in various cancers [56]. Recently, a reduction in *DMBT1* expression has been observed in various cancer types, such as BC, prostate, and gallbladder cancers [57]. In accordance with our previous study, a decrease in the tumor Ki-67 proliferation index (PI) by IHC was observed after eCPMV immunotherapy, indicative of reduced tumor cell cycle proliferation [25].

Among the other upregulated DEGs that promote anti-tumor response, the following genes play important roles: *CLEX7A*, also known as dectin 1, is critical for NK-cell-dependent killing of tumor cells and might bolster anti-tumor immunity [58]; *PRDM1*, which is positively associated with both better cancer prognosis and immune infiltrates, with reduced expression linked to unfavorable prognoses in certain cancer types [59,60]; *CD40LG*, whose CD40/CD40L interaction leads to activation of DCs and contributes to their cytotoxicity against neoplastic cells [61]; *CCRL2*, which is a predictive indicator of potent anti-tumor T cell responses in human cancers [62]; and *IL18R1*, which has been associated with survival prediction in triple-negative breast cancer, primarily through the regulation of CD8^+^ T cells and various subtypes of CD4^+^ T cells [63].

*JAM3*, *LIF*, *CD38*, and *SIGGIR*, upregulated in our study, exert angiogenic and tumor-promoting effects and are linked to unfavorable cancer prognosis [64,65,66,67]. Why these poor prognosis factors are increased in our study needs to be elucidated, but it could be related to the special mechanisms found in IBC/IMC.

### 3.3. Downregulated Immune Pathways and Genes after eCPMV Immunotherapy

The JAK/STAT signaling pathway is involved in tumorigenesis, maintenance, and metastasis in BC. Its downregulation could potentially be a promising therapeutic approach in IBC [68].

In the present study, two genes of the JAK/STAT signaling pathway were significantly reduced after eCPMV treatment: *IL13RA2* and *IL12RB2*. *IL13RA2* plays an important role in cell migration, contributing to tumor progression [69], invasion, and metastasis in several cancers [70]. *IL12RB2* was downregulated after eCPMV treatment. In a lung cancer transplant model assay, it was observed that mice lacking *IL12RB2* developed lung adenocarcinoma [71]. Nevertheless, its role in BC remains unclear.

*TLR5* was found to be the most significantly downregulated DEG compared to other genes (Log2FC = −4.2). This downregulation could be related to the activation of TLR2 and TLR4 by eCPMV treatment [35]. Overexpression of *CD1* is indicative of a negative prognosis in hepatocellular carcinoma [72]. Therefore, the downregulation of this gene following treatment in our study may contribute to improved survival outcomes. Additionally, the downregulation of *S100B*, *IL31RA*, and *CREB5* found here may also promote anti-tumor responses and reduce tumor progression. *S100B*, upregulated in malignant melanomas, has been shown to inhibit the function of the tumor suppressor protein p53 [73]. *IL31RA* has been implicated in BC progression and metastasis [74], while increased expression and activation of *CREB* are associated with tumor growth [75].

Considering the previously published benefits for dogs’ outcome and survival of eCPMV IT-IT, corroborated in the two additional patients of the present study, all these findings provide valuable insights into the immunological effects of in situ eCPMV immunotherapy in IMC. The modulation of immune cell profiles, enhanced anti-tumor immune responses, and improved overall survival collectively underscore the potential of eCPMV as an effective therapeutic strategy for a very-poor-outcome disease. This proof-of-concept study is limited by a small sample size and the genetically diverse nature of the patient groups, which, similar to the previous study, necessitated the comparison of untreated and treated samples not only from the same animal (pre-treatment versus post-treatment) and the absence of randomization. The small and genetically diverse nature of the animal patient groups are potential constraints. While diverse genetic backgrounds are often hailed for mirroring human diversity, it is important to recognize that such limited and diverse groups may pose challenges in terms of drawing unequivocal parallels to the human scenario. However, given the rarity of IMC, the significant results obtained in this study hold great value. Additionally, a more comprehensive transcriptional analysis of neutrophil subpopulations should be pursued to enhance our understanding of their role in the eCPMV therapeutic response.

## 4. Materials and Methods

### 4.1. Patients and Clinical Procedures

The present study is a continuation of our previous research study described in Alonso-Miguel et al. [25], where detailed information about patients, clinical procedures, and outcome are detailed. Here, two additional patients (one treated and one control) have been included, since the recruitment and treatment of animals continued after the publication of the previous study. In total, 12 patients were included in this study (6 eCPMV-treated and 6 eCPMV-untreated patients). Whenever possible, enrolled patients underwent a pretreatment incisional biopsy of the tumor, followed immediately by intratumoral eCPMV injections (Table 3). Briefly, eCPMV IT-IT resulted in tumor shrinkage in all patients by day 14 without adverse events. Although surgery is not recommended in cases of IMC [76], the reduction in tumor size from eCPMV treatment made surgical intervention possible in three of the IMC-treated patients (including the newly treated patient P6). Survival was significantly increased in eCPMV-treated dogs, including the newly enrolled dog P6.

### 4.2. RNA Extraction and NanoString nCounter Analyses

An assessment of tissue preservation and RNA viability was performed prior to RNA extraction from FFPE tissues. Some samples were excluded from further analysis either due to insufficient neoplastic tissue obtained from the incisional biopsy or because the owner did not opt to perform a necropsy (treated dogs P2 and P3; untreated dogs P10 and P11). Ultimately, RNA extraction was performed on 12 tumor samples from untreated patients (n = 8) and eCPMV-treated patients (n = 4). The identification of samples is provided in Table 4.

RNA was extracted from FFPE tissue blocks, where six 5 μm thin sections were taken, resulting in a total thickness of 30 μm scrolls. The samples were then treated with “Deparaffinization Solution” (Qiagen, Werfen, Barcelona, Spain) following the manufacturer’s instructions. Purification of total RNA from FFPE cores was performed using RNeasy FFPE Kit (Qiagen) following the manufacturer’s recommendations. The RNA concentration was quantified using a Nanodrop 2000 Spectrophotometer (Citogen Institution, Zaragoza, Spain).

DV200 (percentage of RNA fragments containing > 200 nucleotides) values were measured using a Bioanalyzer system. The capture and reporter probes contained 50 nucleotides (nt) complementary to the mRNA region of interest. Therefore, the RNA integrity number (RIN) values were not informative for assessing RNA quality for our assays. Instead, we used the DV200 value. Samples with a DV200 value > 30% were considered suitable for subsequent analysis. One IMC pre-treatment sample (P1) was excluded due to low RNA quantity (<20 ng/µL) and poor RNA quality, characterized by high fragmentation or degradation (DV200 < 30%; Supplemental File S1). A standard sample reference was included as an internal control test (Table S1).

The expression levels of immuno-oncology genes in the IMC-treated (n = 4) versus IMC-untreated (control dogs and pre-treatment) RNA samples were quantified using the canine IO panel NanoString nCounter (NanoString Technologies, Seattle, WA, USA). This panel analyzes the gene expression data of 780 genes that reflect cytokines, chemokines, interferon, and checkpoint signaling, complement cascades, immune cell abundance, tumor immunogenicity, inhibitory tumor mechanisms, and stromal factors (Table S1). Nineteen housekeeping genes were included as reference genes and showed homogeneity relative to the quantity/quality of the RNA (Table S2). The RNA was directly hybridized to the reporter and capture probes from the nCounter Canine IO panel. After hybridization, the samples were automatically processed on the nCounter Prep Station, and the captured transcripts were immobilized on the cartridge. The cartridge was then scanned using the nCounter Digital Analyzer to count the barcodes of the reporter probes.

After calculating the background or detection cut of the assay, an average of >700 genes were found to be significantly expressed in all samples (n = 11), except for a post-treatment sample (P2) that showed expression values below the mean (Table S1). This particular sample was taken at necropsy 24 h after the dog died, affecting its RNA quality, and therefore it was excluded from the analyses. A total of 10 samples were included in the downstream analyses.

### 4.3. Transcriptomic Data and Statistical Analyses

The raw data obtained from the NanoString nCounter analyses were normalized and underwent quality control using the nSolver Software v4.0 with the NanoString Advanced Analysis Module v2.0 plugin, following the manufacturer’s instructions. Only registered counts that passed the quality control (QC) parameters were used for subsequent analysis, and the normalized data were then scaled and transformed to log2. Functional analyses included differential gene expression, gene set analysis (GSA), and cell type profiling. Genes were deemed significantly differentially expressed when the *p* values were <0.05. Kyoto Encyclopedia of Genes and Genomes (KEGG) pathway analyses were also performed using the Database for Annotation, Visualization, and Integrated Discovery (DAVID) v6.8 [77] to map clusters of genes involved in common pathways and processes.

Unpaired Student’s *t*-test was used to compare the immune cellular subtyping population between IMC-treated versus IMC-untreated RNA samples. Two-tailed *p* values less than 0.05 were considered statistically significant. Statistical analyses were carried out using IBM SPSS Statistics program (V.25).

## 5. Conclusions

This study provides insights on the transcriptomic changes triggered by eCPMV immunotherapy and their potential influence on the immune response in IMC patients. The significant upregulation and downregulation of specific pathways and genes suggest that eCPMV nanoparticles induce changes in the TME that reflect an anti-tumor immune response, primarily driven by recruiting and activating neutrophils. The use of advanced transcriptomic analysis techniques, such as the Nanostring technology with the canine immune-oncology panel, will contribute to the future development of oncological immune studies in dogs from comparative and veterinary perspectives. These results improve the knowledge of the molecular mechanisms underlying the immune response to eCPMV therapy in IMC. The transcriptomic changes induced by eCPMV treatment and the subsequent amplification of the immune system response highlight the potential of this immunotherapy treatment as a promising therapeutic strategy for canine IMC and a potential future immunotherapy for human IBC patients.

## Figures and Tables

**Table 1:** Up- and downregulated DEGs in female dogs with IMC treated with eCPMV immunotherapy.

Gene Symbol	Log2 Fold Change	Std Error (log2)	*p*-Value	NanoString nCounter Immuno-Oncology Gene Sets
Upregulated				
*CCL17*	5.77	1.1	0.00076	Chemokines, Cytokine and Chemokine Signaling
*DMBT1*	4	1.39	0.0204	
*PTGS2*	3.51	1.02	0.0087	Angiogenesis, Costimulatory Signaling, Cytokine and Chemokine Signaling, Cytokines, Hypoxia, Myeloid Compartment, NF-kB Signaling
*S100A12*	3.06	1.12	0.0257	Cytokine and Chemokine Signaling, Myeloid Compartment
*S100A9*	2.8	1	0.0233	Angiogenesis, Hypoxia
*IL6*	2.62	0.814	0.0122	Angiogenesis, Cytokine and Chemokine Signaling, Hypoxia, Interleukins, JAK-STAT Signaling, Metabolic Stress, PI3K-Akt
*CR2*	2.36	0.847	0.0235	B-Cell Functions, Complement System
*LIF*	2.14	0.805	0.0291	Angiogenesis, Cell Functions, Cytokine and Chemokine Signaling, Cytotoxicity, JAK-STAT Signaling, Myeloid Compartment
*CXCL8*	2.07	0.72	0.0206	Chemokines, Cytokine and Chemokine Signaling, Cytokines, Interleukins, Pathogen Defense, Regulation
*CD40LG*	1.66	0.669	0.0382	Costimulatory Signaling, Cytokine and Chemokine Signaling, Immune Cell Adhesion and Migration, Lymphoid Compartment, NF-kB Signaling, Regulation
*CR1L*	1.43	0.545	0.0304	Complement System
*CCL20*	1.35	0.538	0.036	Chemokines, Cytokine and Chemokine Signaling, Myeloid Compartment
*CLEC7A*	1.17	0.411	0.0212	Immune Cell Adhesion and Migration, Myeloid Compartment
*CD38*	1.11	0.426	0.0316	B-Cell Functions, Hypoxia, Lymphoid Compartment, Regulation, T Cell Functions
*CD4*	0.993	0.323	0.0152	Antigen Presentation, Costimulatory Signaling, Cytokine and Chemokine Signaling, Immune Cell Adhesion and Migration
*FCRL2*	0.989	0.38	0.0316	
*CCRL2*	0.972	0.394	0.0389	Chemokines
*TNFAIP3*	0.891	0.308	0.02	Angiogenesis, Hypoxia, NF-kB Signaling, TNF Superfamily
*JAM3*	0.883	0.352	0.0365	Angiogenesis, Immune Cell Adhesion and Migration
*PRDM1*	0.829	0.299	0.0242	Cell Functions, Epigenetic Regulation
*IL18R1*	0.585	0.25	0.0478	Costimulatory Signaling, Cytokine and Chemokine Signaling, Lymphoid Compartment, NK Cell Functions, T Cell Functions
*CD68*	0.578	0.236	0.0401	Cell Functions
*NFKBIA*	0.573	0.21	0.0257	Apoptosis, Costimulatory Signaling, Cytokine and Chemokine Signaling, NF-kB Signaling
*LY9*	0.534	0.2	0.0284	Costimulatory Signaling, Lymphoid Compartment
*BAX*	0.384	0.152	0.0353	Apoptosis, Cell Cycle, Regulation
*SIGIRR*	0.314	0.13	0.0423	Cytokine and Chemokine Signaling
Downregulated				
*TLR5*	−4.21	1.06	0.0041	TLR
*CD1E*	−2.21	0.882	0.0365	Antigen Processing
*S100B*	−2.02	0.635	0.0129	Cytokine and Chemokine Signaling
*IL13RA2*	−1.77	0.577	0.0153	Chemokines, Cytokine and Chemokine Signaling, JAK-STAT Signaling, T Cell Functions
*CREB5*	−1.7	0.736	0.0494	
*IFGGC1*	−1.32	0.54	0.0406	Macrophage Functions, Myeloid Compartment
*IL12RB2*	−1.25	0.378	0.0106	Cytokine and Chemokine Signaling, Cytokines, Cytotoxicity, JAK-STAT Signaling, Lymphoid Compartment, NK Cell Functions, T Cell Functions
*IL31RA*	−0.884	0.342	0.0323	Cytokine and Chemokine Signaling, Cytokines, Regulation

**Table 2:** Leukocyte subpopulations between IMC samples treated and untreated with eCPMV immunotherapy.

Cell Type	Untreated IMC Dogs	Treated IMC Dogs	*p*-Value *
CD45	10.8 ± 0.4	11.0 ± 0.2	0.192
CD8^+^ T cells	7.0 ± 0.9	7.5 ± 0.4	0.208
Mast cells	6.8 ± 1.4	7.5 ± 1.2	0.248
Neutrophils	7.7 ± 1.6	10.1 ± 1.7	0.031 *
T cells	7.6 ± 0.7	8.3 ± 0.6	0.09
Cytotoxic cells	6.1 ± 0.9	6.6 ± 0.7	0.227
Th1 cells	6.8 ± 0.6	6.5 ± 0.3	0.226
NK CD56dim cells	8.0 ± 0.5	8.2 ± 0.7	0.276
Treg	4.9 ± 0.6	5.6 ± 0.6	0.069

CD45, leucocyte population; CD8^+^ T cells, cytotoxic T cell population; mast cells, mastocytic cell population; neutrophils, neutrophilic population; cytotoxic cells, cytotoxic cell population; T cells, lymphocyte cell population; Th1 cells, T helper cell population; NK CD56dim cells, CD56+ Natural killer (NK) cells; Treg, regulatory T cells; ± denotes standard error; * denotes *p*-value below 0.05.

**Table 3:** Epidemiological and clinicopathological characteristics of eCPMV-treated and -untreated IMC patients.

PATIENT	Age y	Weight kg	Breed	Type	Histo Grade	Histo Type	sdLVI	LNI	Treatments Target Tumor	Therapy	OS Days
eCPMV-treated IMC patients											
P1	11	10.3	Mixed	Primary	III	Special type	Yes	Yes	8	FCT+	174
P2	13.5	25.6	Mixed	Secondary	III	Simple	Yes	Yes	7	FCT+	156
P3	10.7	8.2	Poodle	Secondary	III	Simple	Yes	Yes	2	FCT+	109
P4	10.7	17	Kerry Blue Terrier	Secondary	III	Simple	Yes	Yes	3	FCT+	165
P5	11.9	2.7	Bichon Frise	Secondary	III	Simple	Yes	Yes	2	FCT+	67
P6	13	22.3	German Sheperd	Secondary	III	Simple	Yes	Yes	2	FCT+	104
Untreated IMC patients											
P7	13	26.2	Mixed	Secondary	III	DA	Yes	Yes		FCT	27
P8	14.2	7.6	Maltese	Secondary	III	Simple	Yes	Yes		FCT	40
P9	9.7	10.3	Mixed	Secondary	III	Simple	Yes	Yes		FCT	132
P10	13	9.3	Miniature Schnauzer	Secondary	III	Simple	Yes	Yes		FCT	63
P11	8.9	7.7	Poodle	Secondary	III	Simple	Yes	Yes		FCT	73
P12	8.3	26	German Sheperd	Secondary	III	Simple	Yes	Yes		FCT	14

Age y, age at diagnosis in years; FCT, firocoxib+cyclophosphamide+toceranib; FTC+ indicates that eCPMV-treated dogs received FCT therapy starting after second eCPMV injection until surgery or death; DA, ductal associated; eCPMV, empty cowpea mosaic virus; Histo, histologic; IMC, inflammatory mammary cancer; sdLVI, superficial dermal lymphovascular invasion; LNI, regional lymph node involvement; OS days, overall survival time.

**Table 4:** RNA-IMC samples included in the study.

Patient	Group	D0	Post—TT
P1	IMC—eCPMV	RNA-T1a *	RNA-T1b
P2	IMC—eCPMV	RNA-T2a	RNA-T2b *
P5	IMC—eCPMV	RNA-T3a	RNA-T3b
P6	IMC—eCPMV	RNA-T4a	RNA-T4b
P7	IMC—control	RNA-C1	
P8	IMC—control	RNA-C2	
P9	IMC—control	RNA-C3	
P12	IMC—control	RNA-C4	

Legend. eCPMV: empty cowpea mosaic virus; IMC: inflammatory mammary cancer; RNA-T: treatment group sample; RNA-C: untreated control group sample; D0: biopsy sample before eCPMV inoculation; Post-TT: biopsy sample after eCPMV inoculation. Four samples (n = 4) from untreated control IMC patients (RNA-C1, RNA-C2, RNA-C3, RNA-C4) and samples from IMC-treated patients (RNA-T1, RNA-T2, RNA-T3, RNA-T4) at two different time points: before eCPMV inoculation on day 0 (n = 4) (RNA-T1a, RNA-T2a, RNA-T3a, RNA-T4a) and after treatment (n = 4) (RNA-T1b, RNA-T2b, RNA-T3b, RNA-T4b). * These samples were excluded from the analyses due to poor RNA quality.

## Data Availability

Data available on request from the authors.

## Supplementary Materials

The following supporting information can be downloaded at https://www.mdpi.com/article/10.3390/ijms241814034/s1.

## Abbreviations

APC	antigen-presenting cells
D0	day of first immunotherapy dose
D7	day of second immunotherapy dose
DAVID	Database for Annotation, Visualization, and Integrated Discovery
DAMPs	damage-associated molecular pattern
DCs	dendritic cells
DEGs	differentially expressed genes
eCPMV	empty Cowpea Mosaic Virus
FFPE	formalin-fixed paraffin-embedded
GSA	gene set analysis
IBC	inflammatory breast cancer
IHC	immunohistochemistry
IMC	inflammatory mammary cancer
IL-8	interleukin-8
IO	immuno-oncology
itRECIST	intratumoral Response Evaluation Criteria in Solid Tumors
IT-IT	Intratumoral immunotherapy
KEGG	Kyoto Encyclopedia of Genes and Genomes
MPO	mieloperoxidase
MT	medical therapy
N1/N2	neutrophil subpopulations
NF-κB	nuclear factor-kappaB
NK cells	natural killer cells
Nt	nucleotide
PD-1	programmed cell death 1
PI	proliferation index
P1-12	patients 1-12
QC	quality control
QOL	quality of life
RIN	RNA integrity number
TANs	tumor-associated neutrophils
TLR2/4	Toll-like receptors 2/4
TME	tumor microenvironment
TNF	tumor necrosis factor
Treg	regulatory T cells
VLPs	plant-based virus-like nanoparticles
DEGs Abbreviations
CCL17	CC chemokine ligand 17
DMBT1	deleted in malignant brain tumor 1
PTGS2	prostaglandin-endoperoxide synthase 2
S100A12	S100 Calcium Binding Protein A12
S100A9	S100 calcium-binding protein A9
IL6	interleukin 6
CR2	complement receptor 2
LIF	Leukemia Inhibitory Factor
CXCL8	IL-8 or chemokine (C-X-C motif) ligand 8
CD40LG	CD40 Ligand
CR1L	Complement Receptor 1-Like
CCL20	C-C Motif Chemokine Ligand 20
CLEC7A	C-type lectin domain family 7 member A
CD38	Cluster of Differentiation 38
CD4	CD4 receptor, cluster of differentiation 4
FCRL2	Fc Receptor-Like 2
CCRL2	Chemokine C-C Motif Receptor-Like 2
TNFAIP3	TNF Alpha Induced Protein 3
JAM3	Junctional Adhesion Molecule 3
PRDM1	PR domain zinc finger protein 1
IL18R1	Interleukin 18 Receptor 1
CD68	Cluster of Differentiation 68
NFKBIA	Nuclear Factor Kappa B Inhibitor Alpha
LY9	Lymphocyte Antigen 9
BAX	BCL2 Associated X Protein
SIGIRR	Single Immunoglobulin and Toll-Interleukin 1 Receptor Domain-Containing Protein
TLR5	Toll-like receptor 5
CD1E	Cluster of Differentiation 1E
S100B	S100 calcium-binding protein B
IL13RA2	Interleukin-13 receptor subunit alpha-2
CREB5	cAMP Response Element-Binding Protein 5
IFGGC1	Interferon Gamma-Inducible GTPase Candidate 1
IL12RB2	interleukin 12 receptor, beta 2 subunit
IL31RA	Interleukin 31 Receptor A
